# On the Beneficial Effect of MgCl_2_ as Electrolyte Additive to Improve the Electrochemical Performance of Li_4_Ti_5_O_12_ as Cathode in Mg Batteries

**DOI:** 10.3390/nano9030484

**Published:** 2019-03-26

**Authors:** Marta Cabello, Gregorio F. Ortiz, Pedro Lavela, José L. Tirado

**Affiliations:** Departamento de Química Inorgánica e Ingeniería Química, Instituto Universitario de Investigación en Química Fina y Nanoquímica (IUNAN), Universidad de Córdoba, Campus de Rabanales, Edificio Marie Curie, E-14071 Córdoba, Spain; z22cabbm@uco.es (M.C.); iq1lacap@uco.es (P.L.); iq1ticoj@uco.es (J.L.T.)

**Keywords:** Li_4_Ti_5_O_12_, magnesium batteries, cathodes, MgCl_2_

## Abstract

Magnesium batteries are a promising technology for a new generation of energy storage for portable devices. Attention should be paid to electrolyte and electrode material development in order to develop rechargeable Mg batteries. In this study, we report the use of the spinel lithium titanate or Li_4_Ti_5_O_12_ (LTO) as an active electrode for Mg^2+^-ion batteries. The theoretical capacity of LTO is 175 mA h g^−1^, which is equivalent to an insertion reaction with 1.5 Mg^2+^ ions. The ability to enhance the specific capacity of LTO is of practical importance. We have observed that it is possible to increase the capacity up to 290 mA h g^−1^ in first discharge, which corresponds to the reaction with 2.5 Mg^2+^ ions. The addition of MgCl_2_·6H_2_O to the electrolyte solutions significantly improves their electrochemical performance and enables reversible Mg deposition. Ex-situ X-ray diffraction (XRD) patterns reveal little structural changes, while X-ray photoelectron spectrometer (XPS) (XPS) measurements suggest Mg reacts with LTO. The Ti^3+^/Ti^4+^ ratio increases with the amount of inserted magnesium. The impedance spectra show the presence of a semicircle at medium-low frequencies, ascribable to Mg^2+^ ion diffusion between the surface film and LTO. Further experimental improvements with exhaustive control of electrodes and electrolytes are necessary to develop the Mg battery with practical application.

## 1. Introduction

Magnesium batteries are promising energy storage devices, due to their natural virtues, such as abundance, high theoretical volumetric capacity (3832 mA h cm^−3^), and operational safety [1,2,3,4,5]. However, the development of Mg batteries has been blocked by the lack of proper inorganic cathode materials, which commonly suffer from extremely slow kinetics of the insertion of Mg^2+^ into the intercalation host. Also, the development of suitable electrolytes is a “bottleneck” for the progress of practical Mg batteries.

Electrolytes containing ethereal solvents and organo-magnesium compounds are only partially appropriated for meeting the needs of functional devices in portable electronics and transportation applications [6,7,8]. Electrolytes based on inorganic Mg salts have also been considered, which show significant improvements in terms of stability and corrosion control of the cell components [9,10,11]. The compound δ-MgCl_2_ shows a marked crystallographic disorder, reactivity, and solubility. The structure is built of concatenated MgCl_2_ repeating units, in which the Mg atoms are linked together by chloride bridges [12,13] that provide unconventional properties to the solid. Mg(TFSI)_2_ (TFSI^−^: bis(trifluoromethanesulfonyl)imide) anions), a magnesium analogue of LiTFSI, dissolved in ionic liquids is a common additive in battery electrolytes [14]. Recently, stable and reversible magnesium plating/stripping was reported for Mg(TFSI)_2_ in dimethoxyethane (DME) and Mg(TFSI)_2_ in glyme, when MgCl_2_ or Mg(BH_4_)_2_ or anthracene was added [15,16,17]. The addition of chloride prevents the passivation of the Mg electrode and facilitates the Mg plating/stripping process, as a result of the formation of the binuclear complex, [Mg_2_(μ-Cl)_2_]^2+^, as an intermediate complex [18,19]. Additionally, the electrolyte, composed of magnesium triphenolate borohydride and Mg(TFSI)_2_, displays reversible Mg insertion/de-insertion in the Mo_6_S_8_ Chevrel cathode phase delivering a capacity value of 94 mA h g^−1^ and 96% coulombic efficiency, as reported by Hebié et al. [17]. In these papers, it is claimed that the electrochemical performance of the TFSI-based electrolyte solutions is governed by their purity level. They achieved reversible Mg deposition leading to very high cycling efficiencies with purified DME/Mg(TFSI)_2_/MgCl_2_ [16,17,18,19,20].

Among the other possible candidates to the cathode of Mg batteries, lithium titanate or Li_4_Ti_5_O_12_ (LTO) has been considered here. LTO is a well-known electrode material, with insertion properties useful for Li-ion and Na-ion batteries, that have been already studied [21,22,23,24]. It offers stable discharge plateaus at 1.55 V vs. Li/Li^+^ and 0.8 V vs. Na/Na^+^, which makes it safer and more stable than graphite. Up till now, little attention has been paid to its use in Mg batteries [25,26]. A different scientific approach was introduced by studying Li_4_Ti_5_O_12_ as cathode using hybrid Mg^2+^/Li^+^ electrolytes in Mg batteries [27,28]. The concept of hybrid electrolyte, such as Mg^2+^/Na^+^, was also used for the sodium vanadate compound (β-NaV_6_O_15_) [29]. In both cases, hybrid Mg^2+^/Li^+^ and Mg^2+^/Na^+^ electrolytes could synergistically exploit the advantages of Li^+^, Na^+^ and Mg^2+^. In the former, 0.5 M Mg(BH_4_)_2_ + 1.5 M LiBH_4_ in tetraglyme (TG), and 0.4 M (PhMgCl)_2_–AlCl_3_ + 1.5 M LiBH_4_ in tetrahydrofuran (THF)-based mixed electrolyte, were used as the electrolyte, based solely on Mg salt (0.5 M Mg(BH_4_)_2_ or (PhMgCl)_2_-AlCl_3_), did not exhibit any electrochemical performance.

In the present work, we study the electrochemical reaction of magnesium with LTO. For that purpose, a mixture of Mg(TFSI)_2_ and MgCl_2_·6H_2_O salts in dimetoxyethane (DME) was used. We have found a negligible electrochemical reaction of magnesium with LTO, by using either, Mg(TFSI)_2_ in DME, or MgCl_2_·6H_2_O in DME, separately. Therefore, the freshly prepared electrolyte (TFSI^−^ + Cl^−^) could diminish the strong coulombic interaction between Mg^2+^ and the inorganic framework. Upon the first discharge, the ex-situ XRD and XPS measurements revealed negligible shifting of the *hkl* reflections, and the appearance of Ti^3+^ on the surface of LTO particles, respectively. Mg^2+^ ion diffusion between the surface film and LTO is observed by electrochemical impedance spectra (EIS). The results, which were obtained by allowing water molecules to remain in the inorganic salt, may be useful in comparing with the results obtained by using the anhydrous salt.

## 2. Experimental

Lithium titanate was obtained by a sol-gel route. For the preparation of the precursor gel, 30.7 mL of titanium isopropoxide (purity 97%, Sigma-Aldrich Química S.L., Madrid, Spain) was added to a solution containing 8.13 g of lithium acetate in 19.3 mL of ethanol. The mixture was heated at 100 °C for 14 h under magnetic stirring. The amorphous compound obtained at this step was ground and further annealed at 800 °C in air for 8 h. Manual grinding with lithium acetate, followed by additional annealing at 800 °C in air for 8 h, was needed to remove rutile impurities. 

X-ray diffraction (XRD) patterns were recorded in a Bruker D8 Advance diffractometer (Bruker Española S.A., Madrid, Spain) with a LYNXEYE XE -High-Resolution Energy-Dispersive 1-D Detector and Cu Kα radiation. From line broadening analysis by using Voigt functions, the crystallite size was calculated as the integral breadth-based volume-weighted column height (IB-LVol). The analysis of the chemical state was carried out in an X-ray photoelectron spectrometer (XPS) (SPECS Phobios 150 MCD) provided with Mg Kα source. Powdered samples were placed onto Al foil and subjected to a high vacuum overnight (5 × 10^−9^ mbar). For the ex-situ analysis, the electrode material was transferred to the XPS apparatus under an Ar atmosphere. The binding energy values were referred to the C 1s line of the adventitious carbon located at 284.6 eV. 

The electrochemistry was performed in a three-electrode configuration using LTO as cathode, and Mg foil as anode and reference electrodes. The cells were assembled in an argon filled glove box under controlled O_2_ (2 ppm) and H_2_O (1 ppm) traces. The active material (LTO, 80%) was mixed with PVdF (10%) and carbon black (10%). These components were dispersed in N-methyl-2-pyrrolidone, yielding a homogenous paste which is spread onto a 9 mm Ti foil (for cycling) and carbon paper (for cycling and ex-situ analyses). The electrode was vacuum dried at 120 °C for 2 h. The electrodes were separated by glass fiber disks (GF/A-Whatman) impregnated in the electrolyte solution. The composition of the electrolytes used in this study is the following: a) 0.50 M Mg(TFSI)_2_ + 0.13 M MgCl_2_·6H_2_O in DME (1,2-dimetoxyethane) b) 0.50 M Mg(TFSI)_2_ in DME, and c) 0.13 M MgCl_2_·6H_2_O in DME. The role of water molecules was studied with the following electrolytes: a) 0.50 M Mg(TFSI)_2_ + 0.13 M MgCl_2_ in DME, b) 0.50 M Mg(TFSI)_2_ + 0.13 M MgCl_2_·6H_2_O in DME + 0.5 M H_2_O and c) 0.50 M Mg(TFSI)_2_ + 0.13 M MgCl_2_·6H_2_O in DME + 1.0 M H_2_O. The purity of the reagents was as follows: MgCl_2_·6H_2_O (Sigma Aldrich, ≥99.0%), Mg(TFSI)_2_ (Aldrich, 99%), and 1,2-Dimetoxyethane (Aldrich, 99.5% anhydrous).

The electrochemical impedance spectra were measured by using an SP-150 Biologic apparatus to determine the cell impedance. For this purpose, the three-electrode cells, with LTO as the working electrode, and Mg as counter and reference electrodes, were subjected to a few cycles. After the voltage relaxation pursuing a quasi-equilibrium state, the impedance spectra were measured by perturbing the open circuit voltage with an AC signal of 5 mV from 100 kHz to 0.001 mHz.

## 3. Results and Discussions

### 3.1. Characterization of Li_4_Ti_5_O_12_

**Figure 1:**
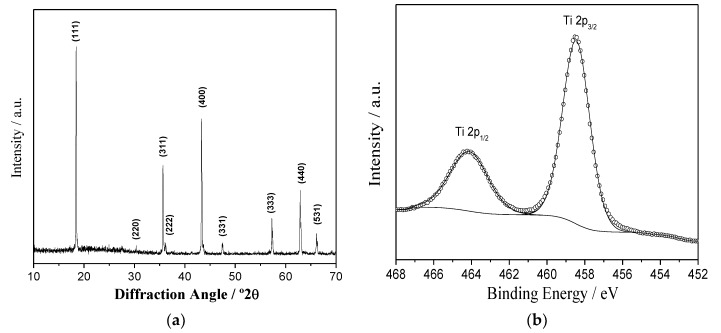

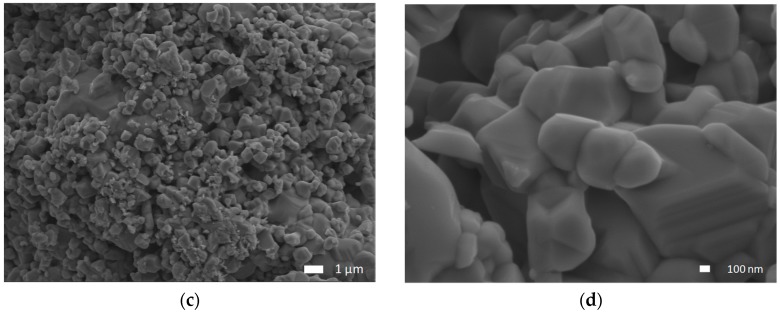
(**a**) X-ray diffraction pattern, (**b**) high-resolution X-ray photoelectron spectroscopy (XPS) spectra showing the Ti2p core levels, and (**c**,**d**) SEM images at different magnifications of LTO sample.

Figure 1a shows the XRD pattern of the Li_4_Ti_5_O_12_. The diffraction peaks are located at 2θ = 18.3°, 35.6°, 36.3°, 43.4°, 47.5°, 57.3°, 62.9°, and 66.2°, and indexed in the *Fd-3m* space group (JCPDS Card No. 49-0207), evidencing the spinel-type structure of Li_4_Ti_5_O_12_. Moreover, the refined lattice parameters of Li_4_Ti_5_O_12_ are coincident with those reported by Ohzuku et al. [30]. The average crystallite size, calculated from *111*, *311* and *400* reflections, ranged between 82.5 and 98.5 nm (Table 1). The chemical composition and surface state for LTO was checked by X-ray photoelectron spectroscopy (XPS). As shown in Figure 1b, the peaks centered at 458.5 and 464.2 eV are assigned to Ti2p_3/2_ and Ti2p_1/2_, which are characteristic of Ti^4+^ in LTO [31]. SEM images at different magnifications revealed that the particle size of the LTO was in the range of 300–500 nm (Figure 1c,d). 

### 3.2. Cyclic Voltammetry with the MgCl_2_-Electrolyte and Control Experiments

**Figure 2:**
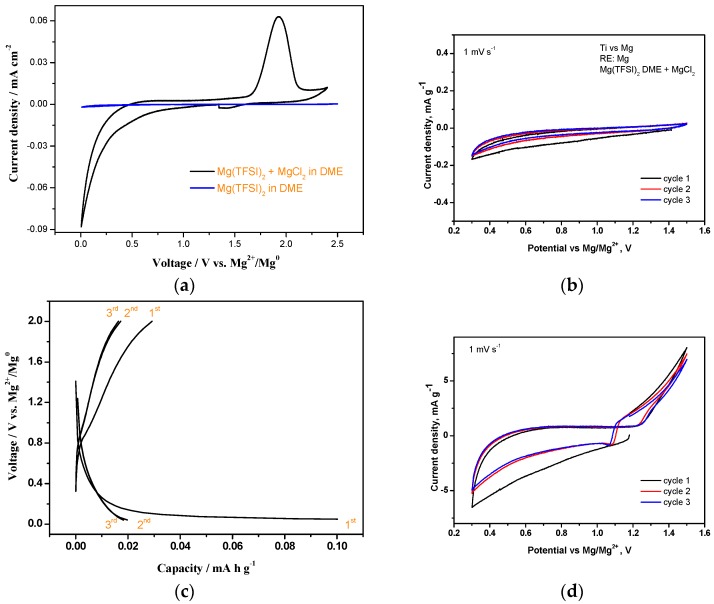
Control experiments without active material, using 0.5 M Mg(TFSI)_2_ + 0.13 M MgCl_2_·6H_2_O, in DME electrolyte versus Mg as reference and counter electrode: (**a**,**b**) Cyclic voltammetry (CV) at 1 mVs^−1^ using different voltage windows (a: 0–2.5 V, b: 0.3–1.5 V). (**c**) Control experiments without active material under galvanostatic cycling at 0.1 C. (**d**) Cyclic voltammetry of LTO with 0.5 M Mg(TFSI)_2_ + 0.13 M MgCl_2_·6H_2_O in DME vs. Mg as reference and counter electrode. Note: The potential is plotted versus the Mg^2+^/Mg voltage of the reference electrode.

**Figure 3:**
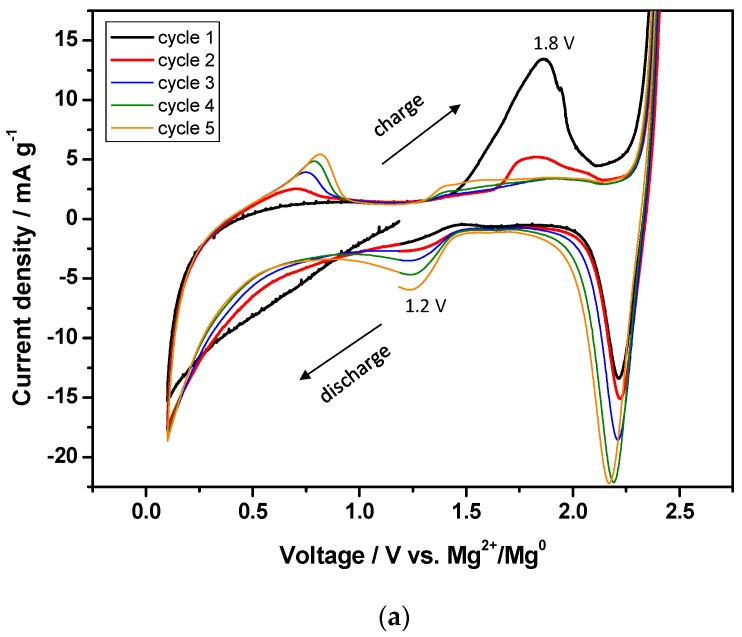

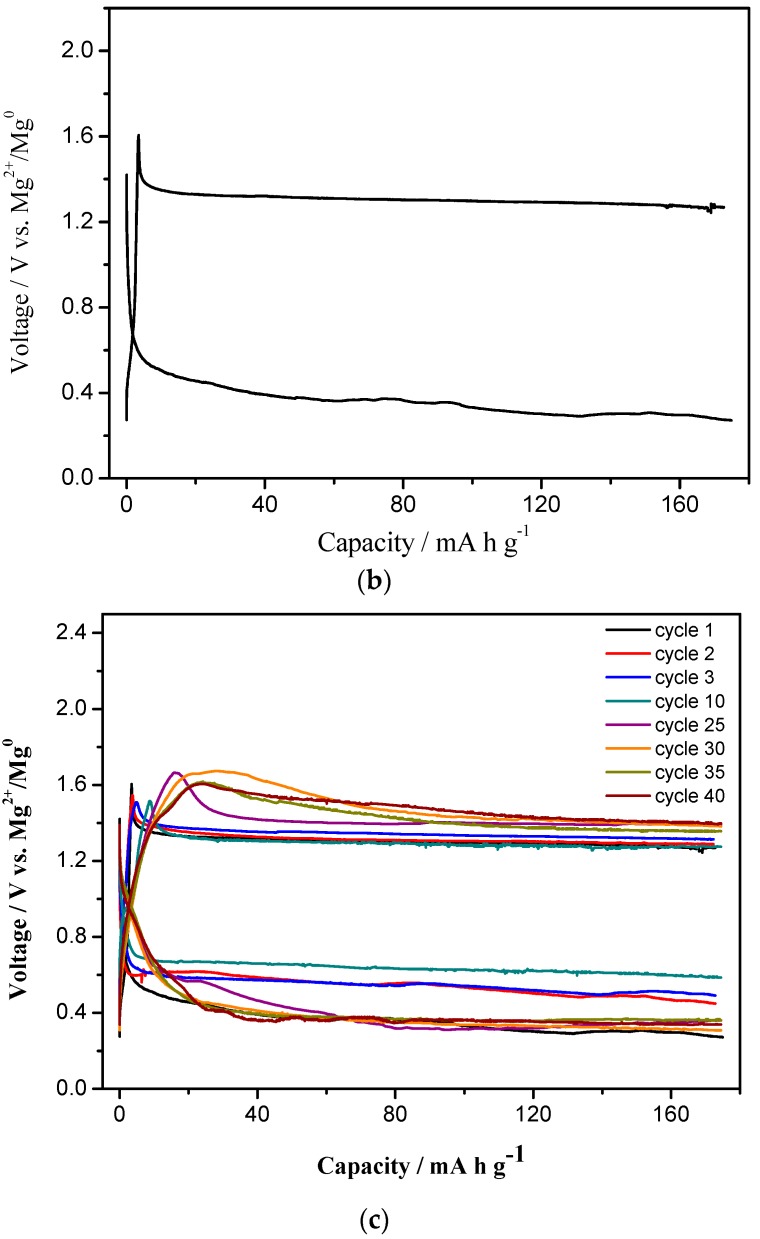
(**a**) Cyclic Voltammetry of LTO with 0.5 M Mg(TFSI)_2_ + 0.13 M MgCl_2_·6H_2_O in DME versus Mg as reference and counter electrode. Galvanostatic discharge/charge curves of LTO sample in a three-electrode Mg cell using: (**b**,**c**) 0.5 M Mg(TFSI)_2_ + 0.13 M MgCl_2_·6H_2_O in DME cycled at 0.1 C.

The cyclic voltammetry of LTO in the Mg cell using 0.5 M Mg(TFSI)_2_ + 0.13 MgCl_2_·6H_2_O in DME electrolyte was recorded using two different voltage windows. No redox peaks were visible between 0.3–1.5 V (Figure 2d). However, the experiments, cycled between 0.1–2.5 V, indicating that the reversible peaks were at 1.2 V on discharge and 0.8 V on charge (Figure 3a). A characteristic large peak starts to appear at about 1.5 V reaching a maximum at 1.8 V. This peak can be related to the activation energy associated with the Mg stripping on the counter electrode. The observation of such peaks may be related to the unusual charge profile observed in the galvanostatic curve of LTO versus metallic Mg in three electrode cell during the charge process (Figure 3b,c). This is probably due to the changes in transport properties arising from the degree of magnesiation as discussed in the next sections.

Control experiments, with no active material, were performed (Figure 2a,b). The range of electrochemical stability of the mixed electrolyte solution Mg(TFSI)_2_-MgCl_2_·6H_2_O in DME was studied by cyclic voltammetry and galvanostatic cycling (Figure 2a,c). The electrolyte solution is stable in the voltage range between 0.0–2.4 V vs. Mg^2+^/Mg^0^. The anodic peak at ca. 1.92 V vs. Mg^2+^/Mg^0^ is ascribed to the stripping of previously electroplated magnesium. The cyclic voltammograms using narrower voltage windows (0.3–1.5 V) did not exhibit any stripping/plating phenomena (Figure 2b). In addition, redox activity in the voltage range between ca. 0.0 and 2.0 V is not observed (Figure 2c) under galvanostatic regime at 0.1 C rate. The comparison of cyclic voltammetry results between Mg(TFSI)_2_ + MgCl_2_·6H_2_O in DME and Mg(TFSI)_2_ in DME reveals that the plating/stripping process is more reversible when using a Mg(TFSI)_2_ + MgCl_2_·6H_2_O mixture than only Mg(TFSI)_2_, thus suggesting the occurrence of synergistic effects when using this combination [32]. 

### 3.3. Extended Discharge and Capacity Retention

**Figure 4:**
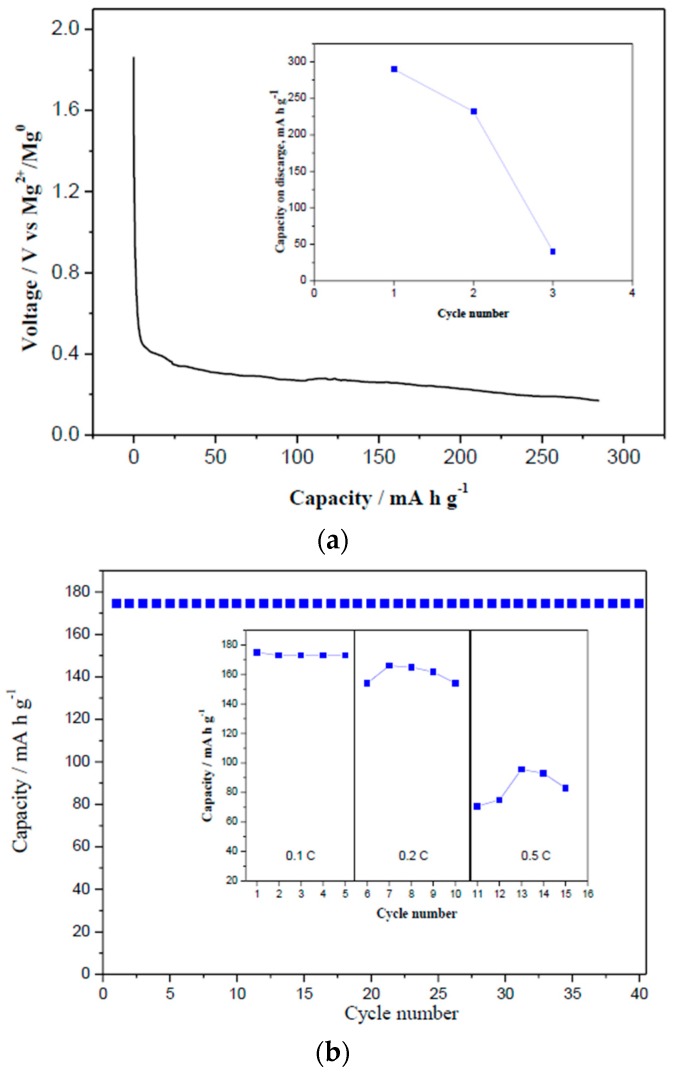
(**a**) Discharge curve of LTO until 290 mA h g^−1^ representing the reaction of magnesium with LTO using 0.5 M Mg(TFSI)_2_ + 0.13 M MgCl_2_·6H_2_O in DME electrolyte. Representative curve obtained for LTO/Mg cell for ex-situ XRD and XPS analysis. The inset in (**a**) represents the capacity retention during 3 cycles. (**b**) Capacity retention of LTO in Mg cell using 0.5 M Mg(TFSI)_2_ + 0.13 MgCl_2_·6H_2_O in DME electrolyte with capacity cut-off. The inset in (b) represents the rate performance with voltage cut-off.

The lithium insertion reaction mechanism in LTO has been previously studied [33]. In general, a structural change from spinel to a rock-salt phase takes place after LTO lithiation. A specific capacity of 175 mA h g^−1^ can be recorded in the 2.5–1 V voltage window versus lithium, which corresponds to the formation of Li_7_Ti_5_O_12_. A mixed valence of Ti^3+^/Ti^4+^ in the latter formula is deduced, meaning that there are two more electrons available for reduction (Li_7_[Ti_2_^4+^Ti_3_^3+^]O_12_). It is possible to reach the Li_9_Ti_5_O_12_ composition by discharging the Li cell to 0 V, delivering a maximum theoretical capacity of 290 mA h g^−1^ [33]. In order to achieve stable capacities in lithium batteries, extremely low voltage limits should be avoided, because the extra lithium-ion intercalation generates a decrease of the ion diffusivity and a large increase of the charge/discharge potential gap [33]. Similarly, our Mg cell, containing LTO as active material, was successfully discharged until 290 mA h g^−1^ (Figure 4a). However, an abrupt capacity fading to <50 mA h g^−1^ was observed after the third cycle (inset of Figure 4a). In order to achieve stable cyclability, the voltage window was limited to the 0.25–1.6 V range vs. Mg^2+^/Mg^0^, in which LTO exhibits good cycling performance (Figure 4b). At the 40th cycle, LTO still delivered 175 mA h g^−1^, corresponding to a capacity retention near 99.9%. These experiments were stopped by limiting Δx to 1.5 Mg. However, the capacity fades to 140 and 80 mA h g^−1^ when cycling at 0.2, and 0.5 C, respectively (inset of Figure 4b). This behavior reflects the slow charge transfer kinetics or the slow diffusion of the Mg^2+^ ions in the LTO lattice or within the electrolyte. Although, further studies are necessary to improve the capacity retention of LTO under high rates, we have found the possibility of enhancing the specific capacity from 175 to 290 mA h g^−1^. The ability to enhance the specific capacity of LTO is useful and offers the opportunity to increase the energy density of Mg cells significantly. 

### 3.4. Effect of MgCl_2_ in Electrolytes on Charge-Discharge Properties (with Capacity Cut-Off)

A comparison of the effects of electrolyte composition on the electrochemical performance of LTO in Mg cells is shown in Figure 3b,c and Figures S1 and S2. The galvanostatic discharge/charge curves show important differences when cycled at 0.1 C rate. LTO hardly delivers any capacity (<5 mA h g^−1^) when using 0.5M Mg(TFSI)_2_ in DME based electrolyte, indicating that Mg^2+^ could not react with LTO (Figure S1). However, the addition of 0.13 M MgCl_2_·6H_2_O improved the electrochemical performance (Figure 3b,c and Figure S2). Thus, the capacity delivered in first discharge is 175 mA h g^−1^. The first discharge plateau is observed between 0.4–0.3 V, and then is shifted to 0.6–0.5 V (vs. Mg^2+^/Mg^0^) for second and successive cycles. However, the charge plateau is observed at about 1.35 V versus Mg^2+^/Mg^0^, during the first and successive cycles. An average potential difference (ΔE) of 0.75 V between charge and discharge was observed. The charge capacity was 174.7 mA h g^−1^ but presented an unusual profile, exhibiting 99.9% efficiency. Higher voltage hysteresis (ca 1.3 V), and similar charge profiles have been observed in TiS_2_ cathodes [34]. A corrosion phenomenon could be discarded because the process is reversible within this voltage window. Tchitchekova et al. did not relate such charge profile to a characteristic redox behavior, but instead to a nucleation activation energy associated with Ca plating on the counter electrode [34]. Wu et al. appreciated Mg^2+^ intercalation and de-intercalation for LTO at 0.35 and 0.95 V, resulting in ΔE = 0.6 V, respectively [25]. Moreover, by decreasing the particle size, a different deintercalation potential, ΔE and reversible capacity were observed. For instance, reversible capacities of 30 and 170 mA h g^−1^ were obtained for particle sizes ranging between 22–27, and 3–4 nm, respectively [25]. In our results, LTO with crystallite size ranging between 82.5–98.5 nm and particle size 300–500 nm reacted when using 0.5 Mg(TFSI)_2_ and 0.13 M MgCl_2_ in DME as electrolyte. Most probably, the different nature of the electrolyte and particle size justifies these observations. 

As far as we know, studies on LTO in dual electrolytes (Mg^2+^ + Li^+^) have only been previously reported in literature [27,28]. The Mg/LTO cell discharges at 0.6 V. However, the performance of the LTO active material should be compared with other earlier studied cathode materials. For instance, the Mg/0.5 M Mg(TFSI)_2_ + 0.07 M anthracene + 0.1 M MgCl_2_ in diglyme/Mo_6_S_8_ cell displayed two plateaus at 1.1 and 0.95 V during cell discharge, corresponding to Mg^2+^ insertion in the inner and outer sites in the Chevrel phase, respectively [17]. During cell charge, two plateaus are also observed at 1.28 and 1.57 V. A capacity value of 80 mA h g^−1^ was recorded upon the first discharge at the C/20 rate [17]. Another example is the Mg/0.5 M Mg(TFSI)_2_ + 0.5M MgCl_2_ in THF electrolyte/Mo_6_S_8_ cell, in which a first discharge capacity of 67 mA h g^−1^ and plateaus at around 0.8 and 1.25 V in discharge and charge, respectively, were observed [15]. 

An outstanding scientific approach proposed by Nam et al. [35] involved the engagement of crystal water existing in the layered structure of MnO_2_ (Birnessite). These water molecules can effectively screen the electrostatic interactions between Mg^2+^ ions and the anions of the host-framework. Indeed, the desolvatation energy penalty can be mitigated since Mg^2+^ ions intercalate in the hydrated form, which suppresses the coulombic repulsion between cations and the host surface [36,37]. In the latter case, the Mg/0.5 M magnesium perchlorate (Mg(ClO_4_)_2_) in acetonitrile with DI water/MnO_2_ cell delivered a large reversible capacity of 231.1 mA h g^−1^ at 2.8 V versus Mg^2+^/Mg [35].

**Figure 5:**
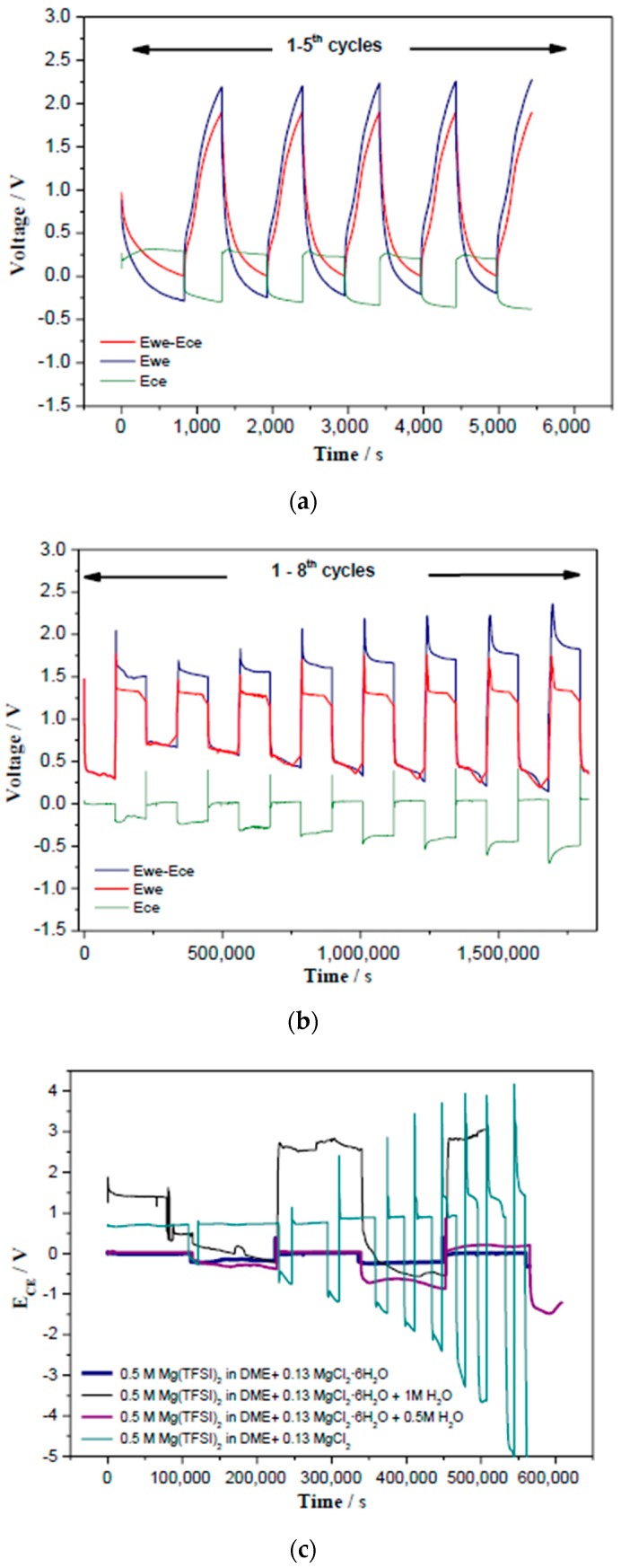
Voltage profiles (E_CE_, E_WE_ and E_WE_-E_CE_) of LTO versus time in a three electrode Mg cell using different electrolytes: (**a**) 0.5 M Mg(TFSI)_2_ in DME, (**b**) 0.5 M Mg(TFSI)_2_ + 0.13 M MgCl_2_·6H_2_O in DME. (**c**) A comparison of E_CE_ versus time of Mg/LTO cell in four different electrolytes containing different amount of water. Note: E_CE_, E_WE_ and E_WE_-E_CE_ refers to the potential of the counter electrode, working electrode and the difference between them, respectively.

In order to distinguish different behaviours in the three electrodes cells, the voltage profiles (E_CE_, E_WE_ and E_WE_-E_CE_) of LTO in a three electrode Mg cell are plotted versus time in Figure 5a,b. Galvanostatic measurements were carried out in Mg cells using 0.5 M Mg(TFSI)_2_ in DME as electrolyte (Figure 5a). A high polarization of about 0.6 V was observed from the first cycle. The over-potential indicates a rather difficult Mg plating/stripping and could be originated from (i) the presence of the native passive layer on Mg electrode and (ii) the reduction of impurities (oxygen, protic species, etc.) [20,38]. 

In contrast, for Mg cells using 0.5 M Mg(TFSI)_2_ + 0.13 M MgCl_2_·6H_2_O in DME as electrolytes, the overpotential was significantly lowered to about 0.15 V in the first cycle, suggesting that the passivating layer was not formed on the Mg foil. The overpotential slightly increased to 0.2, 0.26, 0.31, 0.36, 0.41, 0.43, and 0.48 V from the second to eighth cycle. In spite of this slight increase of polarization (from 0.15 to 0.48 V), the coulombic efficiency keeps around 99.9% on further galvanostatic discharge/charge cycling (Figure 3c and Figure S2a,b). Also, the shape of the voltage profile became rectangular and symmetric, which may correspond to the response of a pure resistance [17]. However, when increasing the water content in the electrolyte (0.5 M H_2_O and 1.0 M H_2_O) we observed a higher polarization in E_CE_ (Figure 5c). Surprisingly, when using anhydrous MgCl_2_, the large polarization is still visible (Figure 5c). In conclusion, the electrolyte containing 0.5 M Mg(TFSI)_2_ in DME + 0.13 M MgCl_2_·6H_2_O exhibited the best electrochemical performance in terms of higher capacity and lower E_CE_ polarization.

### 3.5. Change in the Li_4_Ti_5_O_12_ Lattice by the Charge-Discharge

**Figure 6:**
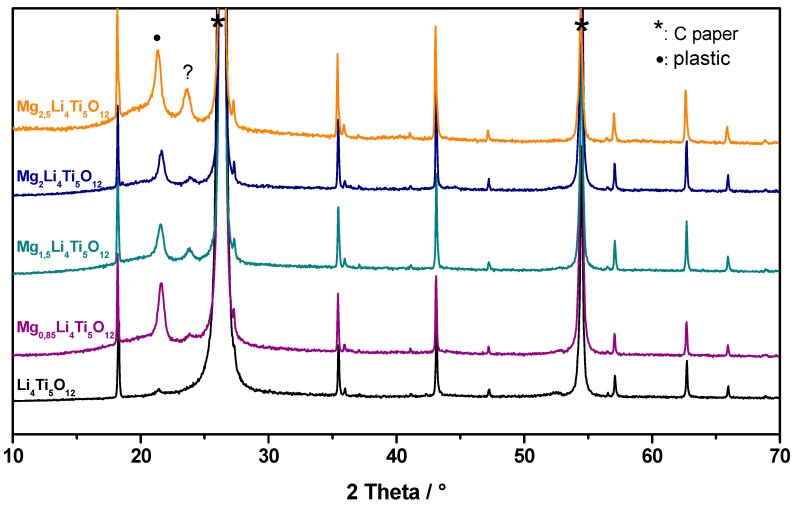

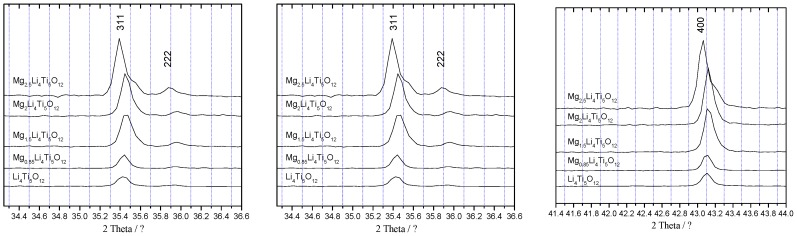
Ex-situ XRD patterns measured during the first discharge in the LTO/Mg cell with 0.5 M Mg(TFSI)_2_ + 0.13 M MgCl_2_·6H_2_O in DME electrolyte. The XRD patterns were recorded at x = 0, 0.85, 1.5, 2, and 2.5 (x in Mg_x_Li_4_Ti_5_O_12_) which correspond to 0, 100, 175, 233 and 290 mA h g^−1^ capacity, respectively. Note: For the sake of clarity, the zoom of the *111, 311, 222 and 400* peaks is represented. The signal of plastic film is markerd with “**·**” symbol. The “?” symbol denotes some unknown peak not ascribable neither to C (or Ti) substrate nor plastic.

In order to understand the structural changes of Li_4_Ti_5_O_12_ during the discharge, in Mg cell with Mg(TFSI)_2_ + MgCl_2_·6H_2_O in DME electrolyte, ex-situ XRD experiments were performed (Figure 6). On discharging to 100, 175, 233 mA h g^−1^ (x = 0.85, 1.5 and 2 in Mg_x_Li_4_Ti_5_O_12_). There were no observable changes in the position of the peaks. The results show that the XRD pattern is insensitive to subtle structural changes owing to the “zero-strain” of LTO. However, on discharging to 290 mA h g^−1^ (x = 2.5 in Mg_x_Li_4_Ti_5_O_12_), we observed a gradual shift of the *111, 311, 222 and 400* reflections to lower angles. The observed d_111_ = 4.880 Å for x = 2.5 is much higher than d_111_ = 4.844 Å for x = 0, which means an increase in lattice cell volume. It is worth noting that the *111, 311, 222* and *400* peaks show asymmetry. Also, the relative intensity of all peaks increased significantly for an x = 2.5 value. These two changes do not agree with previous observations because a voltage limitation was imposed to reach 175 mA h g^−1^ [26,39]. From the structural point of view, the Mg insertion mechanism into LTO appears similar to that reported by Wu et al. [25,26,28]. Therefore, the mechanism of reaction can be written as follows:

Li_4_Ti_5_O_12_ + 1.5 Mg^2+^ + 3 e^−^ ↔ Mg_1.5_Li_4_Ti_5_O_12_ (1)

Mg_1.5_Li_4_Ti_5_O_12_ + 1 Mg^2+^ + 2 e^−^ ↔ Mg_2.5_Li_4_Ti_5_O_12_ (2)

In the rock-salt structure, the (*8a*) positions are vacant, and Li^+^ and Mg^2+^ ions are located at the (*16c*) positions. It worth noting that the (*8a*) and (*16c*) positions are face sharing [30]. Most probably, the unusual discharge-charge profile of LTO in Mg cells (shown in Figure 3b,c) is due to changes in the transport properties arising from the degree of magnesiation (state of discharge). Indeed, it was previously established that these transport properties will have severe implication in cell kinetics, and asymmetric charge-discharge profiles have also been observed for LTO in liquid electrolytes. This asymmetry is attributed to the change of ionic conductivity during cycling. A core-shell model of the phase transition and a solid solution model were proposed [40,41,42]. 

### 3.6. Changes in the Oxidation State by XPS

**Figure 7:**
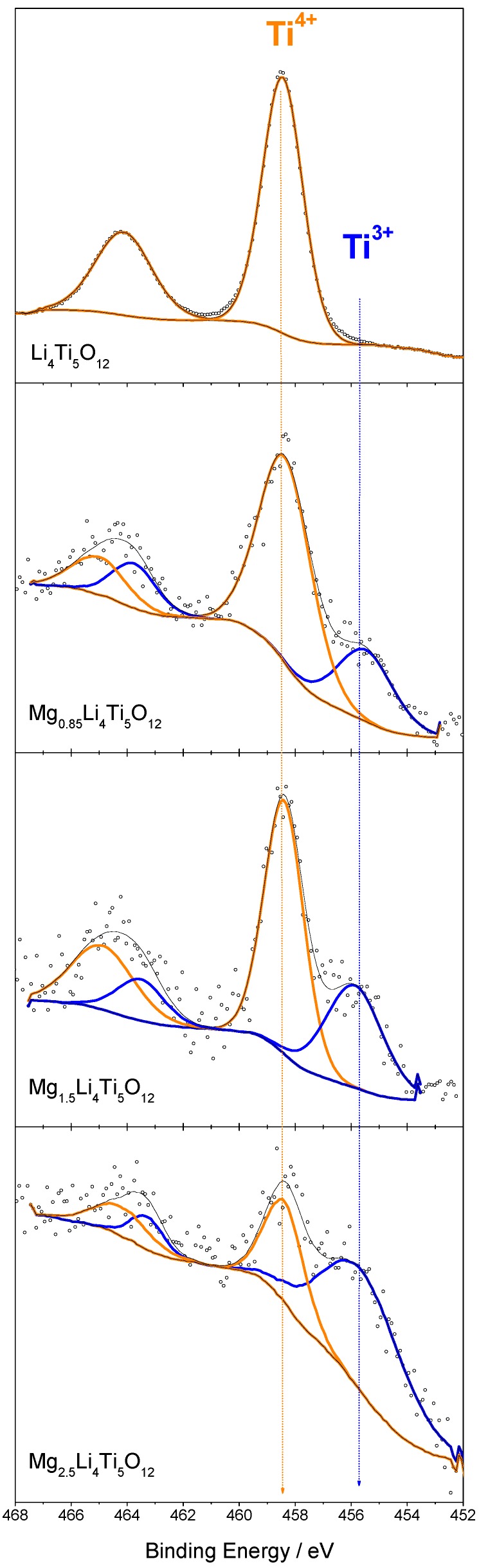
Ex-situ XPS spectra of LTO electrodes electrode in 0.50 M Mg(TFSI)_2_ + 0.13 M MgCl_2_·6H_2_O in DME as electrolyte at x = 0, 0.85, 1.5, and 2.5 (x in Mg_x_Li_4_Ti_5_O_12_) which correspond to 0, 100, 175 and 290 mA h g^−1^ capacity, respectively.

Recently, the XPS technique has been used to investigate changes in the surface of titanium during the insertion reaction of alkali metals in LTO [26,43,44,45]. In this work, ex-situ XPS experiments were performed for Mg_x_Li_4_Ti_5_O_12_ (with x = 0, 0.85, 1.5 and 2.5). Figure 7 shows the XPS spectra in the Ti2p region. The pristine sample shows 458.5 eV binding energies at the Ti2p_3/2_ peak, which are typical of Ti^4+^. When discharging to x = 0.85, a new signal at 455.6 eV is observed. This new signal at lower binding energy confirmed the formation of Ti^3+^ and the quantitative analysis by peak-profile fitting showed 21.3% of Ti^3+^. At x = 1.5 and 2.5, the contribution of the Ti^3+^ signal increased up to 35.6% and 66.4% (Table 1). This is a clear evidence to prove titanium reduction to Ti^3+^ in LTO. However, the Ti^4+^ signal was still present in the fully magnesiated sample (Mg_2.5_Li_4_Ti_5_O_12_), most probably because the cell was charged and discharged in a non-equilibrium state and Mg^2+^ was not migrating fast enough to ensure the full charge transfer to the entire sample. This fact is commonly observed in related ex-situ experiments. Similarly, the Ti^3+^ content (66.4%) detected by XPS when the Mg/LTO cell is discharged to a nominal x = 2.5 represents only x = 1.65 in the intercalation compound. However, the intensity of the Ti^3+^ signal is much higher than that reported for LTO in Mg batteries [26], or in Li batteries [43,44,45]. The observed binding energies of Ti2p_3/2_ and Ti2p_1/2_ values indicated that the oxidation state of Ti-cations in the fully-discharged electrode can be assigned to Ti^4+^ and Ti^3+^. These results evidenced the electrochemical reaction of magnesium with LTO. 

### 3.7. Electrochemical Impedance Spectroscopy

**Figure 8:**
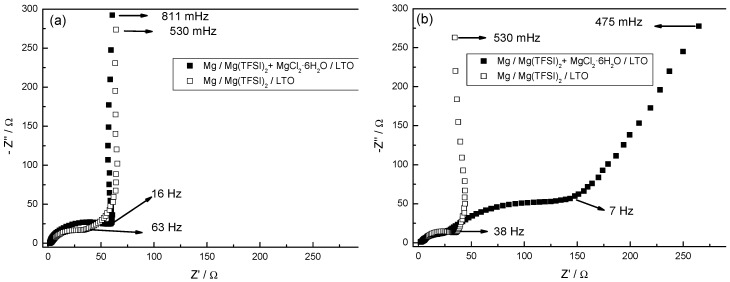

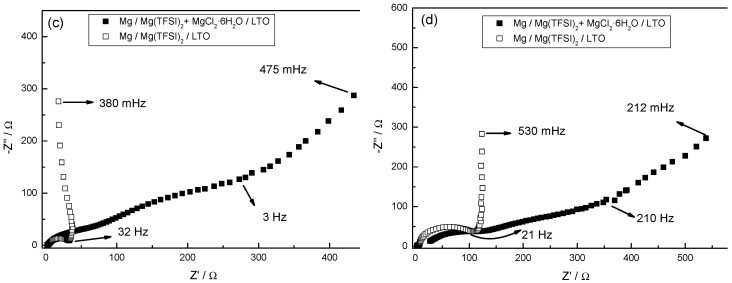
Nyquist plots of LTO electrode in Mg cells using two different electrolytes recorded at: (**a**) open circuit voltage (OCV), and after (**b**) first, (**c**) second and (**d**) fifth discharge. Rate: C/10. Note that the closed square symbols correspond to Mg/0.50 M Mg(TFSI)_2_ + 0.13 M MgCl_2_·6H_2_O in DME/LTO cell, while the open square symbols correspond to Mg/0.50 M Mg(TFSI)_2_ in DME/LTO cell.

Electrochemical impedance spectroscopy is an excellent technique to study the kinetic response of electrode materials. It determines the resistance at the interphase between the working electrode and the electrolyte. Figure 8 shows the Nyquist diagrams of LTO electrode in Mg cells using 0.50 M Mg(TFSI)_2_ in DME with and without 0.13 M MgCl_2_·6H_2_O. These plots reveal different components of the cell resistance by fitting the spectra to the following equivalent circuit: (R_1_+(R_2_/Q_2_)+(R_3_/Q_3_+C_3_)+W_3_). R_1_ refers to the ohmic drop at the electrolyte; R_2_ is assigned to the migration of Mg ions through the surface film into the LTO particles and is calculated from the semicircle at high frequencies (this semicircle being invariant with potential). The charge transfer resistance (R_3_) can be calculated from the semicircle at medium-low frequencies and is ascribed to the transfer of magnesium through the film-mass interface coupled with interfacial capacitance and is potential-dependent. Q, W and C elements label constant phase element, Warburg impedance, and capacitor, respectively. The spectra of the different Mg cells, recorded at open circuit voltage (OCV), exhibit only one semicircle along the high to low frequency values (530 and 811 mHz for Mg/0.5 M Mg(TFSI)_2_ + MgCl_2_·6H_2_O/LTO and Mg/0.5M Mg(TFSI)_2_/LTO, respectively). After the first discharge, the impedance spectrum of the Mg/0.5 M Mg(TFSI)_2_ + MgCl_2_·6H_2_O/LTO cell presents two defined semicircles and a Warburg element (slope of 45°), while the Mg/0.5 M Mg(TFSI)_2_/LTO cell shows a similar profile to that at OCV. The observation of a second semicircle, in medium-low frequencies, represents the existence of a charge transfer resistance. This charge-transfer resistance is undoubtedly assigned to the migration of Mg^2+^ ions between the surface film and the LTO coupled with interfacial capacitance, followed by magnesium ion diffusion in the bulk particle. The R_3_ values obtained in the first, second, and fifth discharge were 62, 130, and 150 Ω·cm^2^, respectively. Curiously, the R_2_ values increased from 13.2 Ω·cm^2^ in the first cycle to 45 Ω cm^2^ in the fifth cycle. The data presented here allow us to infer the positive effects of MgCl_2_·6H_2_O, by facilitating the diffusion of magnesium through the active material, in contrast to a MgCl_2_·6H_2_O-free electrolyte. 

The impedance of magnesium metal anodes is several orders of magnitude higher than in the insertion anodes of lithium-ion commercial cells. Currently, testing of new cathode materials in full cells is often vulnerable to the high impedance exhibited by the magnesium metal anode, which may reach 1 MΩ·cm^2^ [46]. The results presented here are useful to design scientific strategies for minimizing the impedance in Mg batteries.

## 4. Conclusions

In summary, we explored the properties of LTO electrodes for Mg batteries by using different electrolyte compositions. Using a fresh solution of 0.5 M Mg(TFSI)_2_ + 0.13 M MgCl_2_·6H_2_O in DME, the first discharge and charge profile displayed a plateau between 0.4–0.3 V, and 1.35 V Mg^2+^/Mg^0^, respectively. Then, the potential was maintained at 0.6–0.5 V on further discharges. Under these conditions, we obtained 175 and 290 mA h g^−1^ capacities, which correspond to the formation of Mg_1.5_Li_4_Ti_5_O_12_, and Mg_2.5_Li_4_Ti_5_O_12_, respectively. The galvanostatic profiles exhibited a high polarization. Most probably, from an industrial point of view the reported results are not attractive. However, this work attempts to validate a proof of concept of rechargeable magnesium batteries. 

Although further studies are necessary to improve the capacity retention of LTO over a large number of cycles, we have addressed the possibility to enhance the specific capacity from 175 to 290 mA h g^−1^. The ability to enhance significantly the specific capacity of LTO is useful and offers the opportunity to increase the energy density of full cells. The ex-situ XRD patterns are insensitive to subtle structural changes from x = 0–1.5, while for x = 2.5 a shift of the *hkl* reflections was recorded. Ex-situ XPS spectra evidenced changes in the oxidation state of titanium. Therefore, signals of Ti^3+^ (66.4%) and Ti^4+^ (33.6%) at the end of the discharge were obtained. While the charge transfer resistance for Mg/LTO cell without MgCl_2_·6H_2_O additive was not quantifiable, the surface film and the charge transfer resistance for Mg/LTO cell with MgCl_2_·6H_2_O additive were 13.2 and 62 Ω·cm^2^, respectively. This study confirms the electrochemical activity of LTO towards magnesium in a Mg(TFSI)_2_ + MgCl_2_·6H_2_O-based electrolyte.

## Figures and Tables

**Table 1:** Average crystallite size, unit cell parameters and quantitative analysis by peak-profile fitting for lithium titanate or Li_4_Ti_5_O_12_ (LTO) electrodes before and after magnesiation.

	Li_4_Ti_5_O_12_	Mg_0.85_Li_4_Ti_5_O_12_	Mg_1.5_Li_4_Ti_5_O_12_	Mg_2_Li_4_Ti_5_O_12_	Mg_2.5_Li_4_Ti_5_O_12_
IB-LVol_(111)_/nm	82.5	70.8	57.9	74.8	60.1
IB-LVol_(311)_/nm	98.5	78.1	58.4	69.7	66.6
IB-LVol_(400)_/nm	98.0	67.7	63.7	76.0	64.3
a/Å	8.325(2)	8.387(1)	8.387(2)	8.389(2)	8.398(2)
V/Å^3^	577.02(1)	590.16(1)	570.09(2)	590.40(1)	592.47(1)
Ti^4+^/%	100	78.7	64.4	-	33.6
Ti^3+^/%	0	21.3	35.6	-	66.4

## Supplementary Materials

The following are available online at https://www.mdpi.com/2079-4991/9/3/484/s1, Figure S1. Galvanostatic discharge/charge curves of LTO sample in a three-electrode Mg cell using 0.5 M Mg(TFSI)_2_ in DME. Figure S2. Galvanostatic discharge/charge curves of LTO sample in a three-electrode Mg cell using 0.5 M Mg(TFSI)_2_ + 0.13 M MgCl_2_·6H_2_O in DME electrolyte in different current collector: (a–c) Ti foil and (d) C paper.
