# Supplementary material for: On the Beneficial Effect of MgCl2 as Electrolyte Additive to Improve the Electrochemical Performance of Li4Ti5O12 as Cathode in Mg Batteries
Source: Nanomaterials (Basel). 2019 Mar 26;9(3):484. doi: 10.3390/nano9030484 (PMC6474089; doi:10.3390/nano9030484)
Supplement: Supplementary file 1 [file nanomaterials-09-00484-s001.pdf]

Supporting Information

On the beneficial effect of $MgCl_2$ as electrolyte additive to improve the electrochemical performance of $Li_4Ti_5O_{12}$ as cathode in Mg Batteries

Marta Cabello, Gregorio F. Ortiz*, Pedro Lavela and José L. Tirado

[a]*Departamento de Química Inorgánica e Ingeniería Química, Instituto Universitario de Investigación en Química Fina y Nanoquímica (IUNAN), Universidad de Córdoba, Campus de Rabanales, Edificio Marie Curie, E-14071 Córdoba, Spain*

**Corresponding author, email: q72maorg@uco.es*

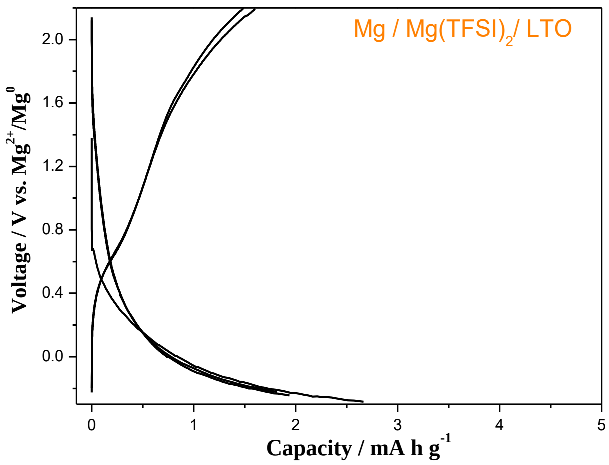

Figure S1. Galvanostatic discharge/charge curves of LTO sample in a three-electrode Mg cell using 0.5 M $Mg(TFSI)_2$ in DME.

Supporting Information

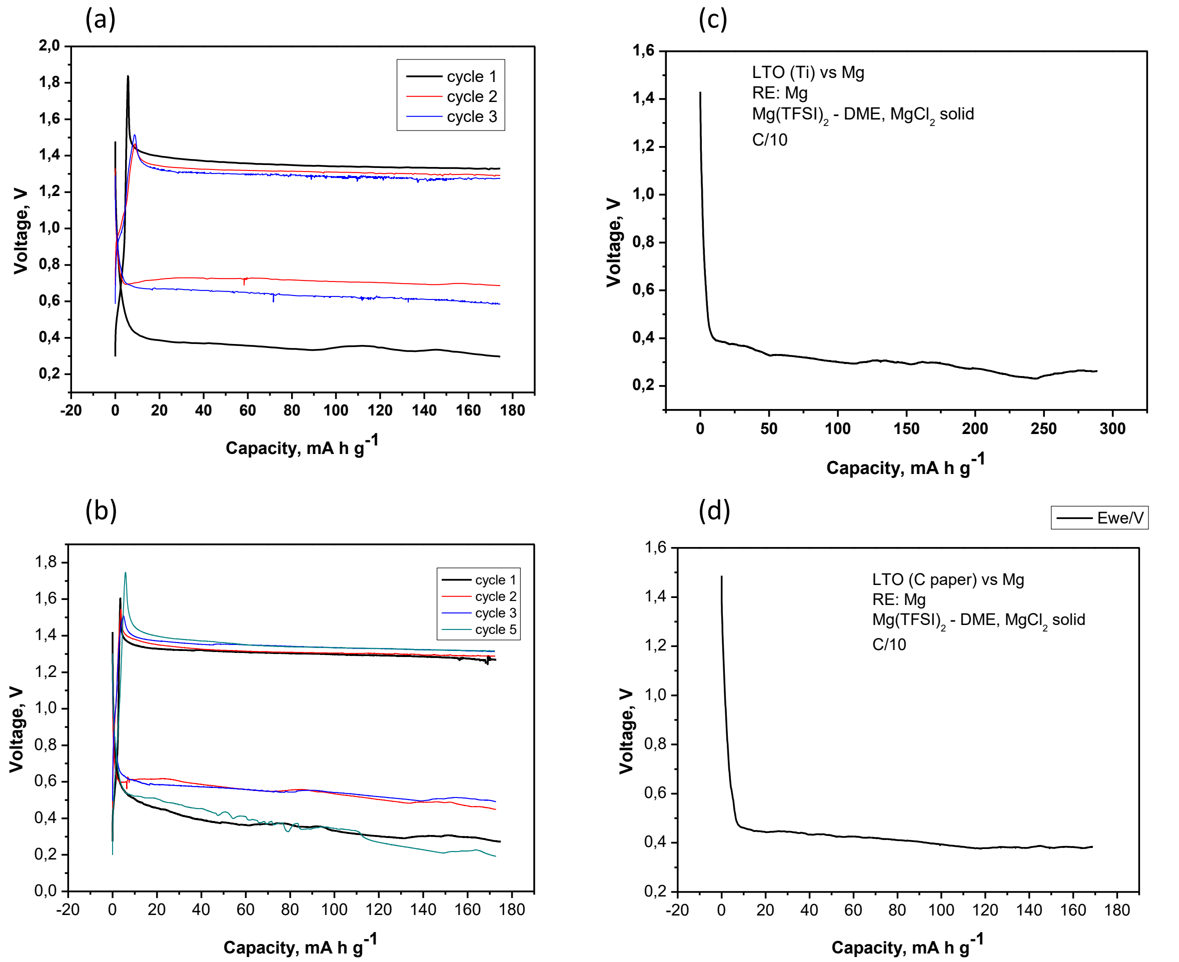

Figure S2. Galvanostatic discharge/charge curves of LTO sample in a three-electrode Mg cell using 0.5 M $Mg(TFSI)_2$ + 0.13 M $MgCl_2 \cdot 6H_2O$ in DME electrolyte in different current collector: (a-c) Ti foil and (d) C paper.
